# Automated Multiclass Classification of Spontaneous EEG Activity in Alzheimer’s Disease and Mild Cognitive Impairment

**DOI:** 10.3390/e20010035

**Published:** 2018-01-09

**Authors:** Saúl J. Ruiz-Gómez, Carlos Gómez, Jesús Poza, Gonzalo C. Gutiérrez-Tobal, Miguel A. Tola-Arribas, Mónica Cano, Roberto Hornero

**Affiliations:** 1 Biomedical Engineering Group, E.T.S.I. de Telecomunicación, Universidad de Valladolid, 47011 Valladolid, Spain; 2 Instituto de Investigación en Matemáticas (IMUVA), Universidad de Valladolid, 47011 Valladolid, Spain; 3 Instituto de Neurociencias de Castilla y León (INCYL), Universidad de Salamanca, 37007 Salamanca, Spain; 4 Servicio de Neurología, Hospital Universitario Río Hortega, 47012 Valladolid, Spain; 5 Servicio de Neurofisiología Clínica, Hospital Universitario Río Hortega, 47012 Valladolid, Spain

**Keywords:** Alzheimer’s disease, mild cognitive impairment, electroencephalography (EEG), spectral analysis, nonlinear analysis, multiclass classification approach

## Abstract

The discrimination of early Alzheimer’s disease (AD) and its prodromal form (i.e., mild cognitive impairment, MCI) from cognitively healthy control (HC) subjects is crucial since the treatment is more effective in the first stages of the dementia. The aim of our study is to evaluate the usefulness of a methodology based on electroencephalography (EEG) to detect AD and MCI. EEG rhythms were recorded from 37 AD patients, 37 MCI subjects and 37 HC subjects. Artifact-free trials were analyzed by means of several spectral and nonlinear features: relative power in the conventional frequency bands, median frequency, individual alpha frequency, spectral entropy, Lempel–Ziv complexity, central tendency measure, sample entropy, fuzzy entropy, and auto-mutual information. Relevance and redundancy analyses were also conducted through the fast correlation-based filter (FCBF) to derive an optimal set of them. The selected features were used to train three different models aimed at classifying the trials: linear discriminant analysis (LDA), quadratic discriminant analysis (QDA) and multi-layer perceptron artificial neural network (MLP). Afterwards, each subject was automatically allocated in a particular group by applying a trial-based majority vote procedure. After feature extraction, the FCBF method selected the optimal set of features: individual alpha frequency, relative power at delta frequency band, and sample entropy. Using the aforementioned set of features, MLP showed the highest diagnostic performance in determining whether a subject is not healthy (sensitivity of 82.35% and positive predictive value of 84.85% for HC vs. all classification task) and whether a subject does not suffer from AD (specificity of 79.41% and negative predictive value of 84.38% for AD vs. all comparison). Our findings suggest that our methodology can help physicians to discriminate AD, MCI and HC.

## 1. Introduction

Dementia due to Alzheimer’s disease (AD) is a progressive neurodegenerative disorder associated with cognitive, behavioral and functional alterations. AD prevalence increases exponentially with age, from 1% in people between 60 and 64 years up to 38% in people over 85 years [1]. Since AD is increasingly being recognized as a modern epidemic, growing efforts have been devoted to exploring its underlying brain dynamics. Despite the considerable progress made to understand AD pathophysiology, a better characterization of its early stages is still required [1]. Mild cognitive impairment (MCI) subjects exhibit a memory impairment beyond what would be expected for their age, but do not fully accomplish the criteria for dementia diagnosis [2]. In this regard, further research is essential to identify incipient AD, since subjects with MCI have high risk of developing it [3]. Recent studies estimated that the conversion rate from MCI to AD is approximately 15% per year [4], whereas this rate is only 1–2% from global population [1]. Despite the fact that current pharmacological treatments and non-pharmacological therapies are not able to heal AD or MCI, an early diagnosis is still crucial since these are more effective in the first stages of the dementia [5].

Several neuroimaging techniques have been used during the last decades with the aim of distinguishing AD and MCI patients from cognitively healthy control (HC) subjects: functional magnetic resonance imaging (fMRI), positron emission tomography (PET), magnetic resonance spectroscopy, electroencephalography (EEG), and magnetoencephalography (MEG), among others [6]. PET and fMRI show a good structural accuracy, but both offer a limited temporal resolution. By contrast, EEG and MEG are non-invasive techniques with high temporal resolution, allowing for studying the dynamical processes involved in the regulation of complex functional brain systems [7]. Particularly, EEG is widely used due to its portability, low cost, and availability. Moreover, EEG has already shown its usefulness to characterize brain dynamics in AD and MCI [7,8,9,10,11,12,13,14].

The abnormalities that AD and MCI elicit in EEG activity have been traditionally analyzed using simple signal processing methods, such as spectral techniques [13,14]. Spectral analyses seem to discriminate AD and MCI patients from HC subjects through a power increase in low frequency bands, as well as a decrease in higher frequencies [13,14]. Since the mid 1990s, nonlinear analysis techniques have also been widely used in order to provide complementary information to spectral measures [10]. Previous studies suggested a more regular EEG activity for AD and MCI patients when compared to HC subjects [11,14]. Other authors reported a decrease of variability and complexity as the disease worsens [7,8,9,12]. However, almost all these studies only applied one or a few methods to partially characterize the brain dynamics in AD and MCI.

The main objective of this study is to evaluate the diagnostic usefulness of an EEG-based methodology by means of different multiclass classifiers: logistic discriminant analysis (LDA), quadratic discriminant analysis (QDA) and multi-layer perceptron neural network (MLP). We hypothesize that the combination of spectral measures and nonlinear methods can be useful to help in AD and MCI diagnosis. For this reason, our proposed methodology is based on both frequency (spectral features) and time domain (nonlinear features) analyses applied to EEG recordings. However, this exhaustive characterization of EEG may lead to obtaining redundant features sharing similar information. In order to avoid this issue, an automatic feature selection stage based on the fast correlation-based filter (FCBF) is followed [15]. Finally, a classification approach is also conducted. Previous studies performed a binary classification approach facing AD vs. HC, MCI vs. HC and AD vs. MCI [16,17,18,19,20]. Only McBride et al. reported a three-way classification, but via binary classifiers [21]. Additionally, their approach was validated through a leave-one-out cross-validation procedure, leading to multiple models. By contrast, our proposal focuses on building a single multiclass model to determine the group for each subject. This is an essential feature for a simplified screening protocol in the future. Afterwards, the group for each subject was settled with a trial-based majority vote procedure, as proposed in previous studies involving early AD recognition [22].

## 2. Materials and Methods

### 2.1. Subjects

EEG data were recorded from 111 subjects: 37 AD patients, 37 MCI patients, and 37 elderly HC subjects. Patients with dementia or MCI due to AD were diagnosed according to the clinical National Institute on Aging and Alzheimer’s Association (NIA-AA) criteria, whereas HC were elderly subjects without a cognitive impairment and with no history of neurological or psychiatric disorder [23]. Inclusion and exclusion criteria for each group can be found in our previous study [20].

All participants and patients’ caregivers were informed about the research background and the study protocol. Moreover, all of them gave their written informed consent to be included in the study. The Ethics Committee at the Río Hortega University Hospital (Valladolid, Spain) endorsed the study protocol, according to The Code of Ethics of the World Medical Association (Declaration of Helsinki).

### 2.2. EEG Recording

Five minutes of spontaneous EEG activity were recorded using a 19-channel EEG system (XLTEK^®^, Natus Medical, Pleasanton, CA, USA). Specifically, EEG activity was acquired from Fp1, Fp2, Fz, F3, F4, F7, F8, Cz, C3, C4, T3, T4, T5, T6, Pz, P3, P4, O1, and O2, at a sampling frequency of 200 Hz. Subjects were asked to stay in a relaxed state, awake, and with closed eyes during EEG acquisition. During the recording procedure, EEG traces were visually monitored in real time, and muscle activity was identified to avoid high-frequency noise. Additionally, independent component analysis (ICA) was performed to minimize the presence of oculographic, cardiographic, and myographic artifacts [7]. Afterwards, EEG signals were digitally filtered using a finite impulse response filter designed with a Hamming window between 1 and 70 Hz and a notch filter to remove the power line frequency interference (50 Hz, Butterworth filter). Finally, an experienced technician selected artifact-free epochs of 5-s by visual inspection.

We randomly divided our EEG database into training and test sets. The training set was formed by: 20 AD patients (45.85 ± 8.36 trials per subject, mean ± standard deviation, SD), 20 MCI subjects (46.85 ± 10.68 trials per subject) and 20 HC subjects (45.60 ± 7.93 trials per subject). The recordings not selected for the training set were assigned to the test set: 17 AD patients (44.53 ± 10.10 trials per subject), 17 MCI subjects (49.82 ± 8.29 trials per subject) and 17 HC subjects (44.24 ± 7.81 trials per subject). No statistically significant differences were found in age (*p*-value > 0.05, Kruskal–Wallis test) and gender (*p*-value > 0.05, chi-squared test) among AD, MCI, and HC groups. Table 1 shows relevant socio-demographic and clinical data for each group.

### 2.3. Methods

**Figure 1:**
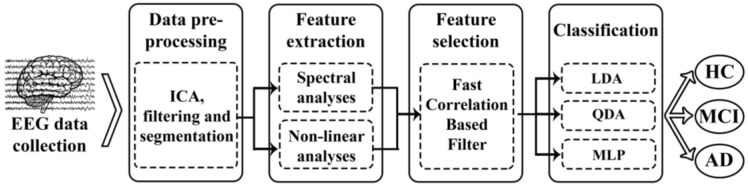
Block diagram of the steps followed in the EEG analysis: data collection, pre-processing, feature extraction, feature selection and classification.

The methodology followed in this study is represented in Figure 1. After EEG-signal recording and data pre-processing, both spectral and nonlinear features were computed. Then, FCBF was applied to the training set to automatically select an optimum set of features. Finally, three different multiclass classification approaches (LDA, QDA, and MLP) were adopted to settle the group for each trial and subject.

#### 2.3.1. Feature Extraction

##### Spectral Analysis

A typical approach to characterize electromagnetic brain recordings is based on the analysis of their spectral content [24,25,26]. Spectral parameters are based on the normalized power spectral density in the frequency band of interest (*PSD_n_*). In this request, the following spectral parameters have been calculated from the *PSD_n_*: relative power (*RP*), median frequency (*MF*), individual alpha frequency (*IAF*), and spectral entropy (*SE*).

- *RP* represents the relative contribution of different frequency components to the global power spectrum. *RP* is more appropriate than absolute power to analyze EEG data, as *RP* provides independent thresholds from the measurement equipment and lower inter-subject variability [27]. *RP* is obtained by summing the contribution of the desired spectral components:
  (1) $$RP\left(f_{1},f_{2}\right)=\sum_{f_{1}}^{f_{2}}PSD_{n}\left(f\right),$$
  where $f_{1}$ and $f_{2}$ are the low and the high cut-off frequencies of each band, respectively.
  In this study, *RP* was calculated in the conventional EEG frequency bands: delta (*δ*, 1–4 Hz), theta (*θ*, 4–8 Hz), alpha (*α*, 8–13 Hz), beta-1 (*β*_1_, 13–19 Hz), beta-2 (*β*_2_, 19–30 Hz) and gamma (*γ*, 30–70 Hz).
- *MF* offers an alternative way to quantify the spectral changes of the EEG, and it is a simple index that summarizes the whole spectral content of the *PSD_n_*. *MF* is defined as the frequency that comprises 50% of the *PSD_n_* power:
  (2) $$\sum_{1Hz}^{MF}PSD_{n}\left(f\right)=0.5\sum_{1Hz}^{70Hz}PSD_{n}\left(f\right).$$
  Previous studies suggested that *MF* provides a better performance for the characterization of brain activity than mean frequency, whose original definition is based on the computation of the spectral centroid [28].
- *IAF* evaluates the frequency at which the maximum alpha power is reached. Alpha oscillations are dominant in the EEG of resting normal subjects, with the exception of irregular activity in the delta band and lower frequencies. This issue involves that the *PSD* displays a peak around the alpha band. The *IAF* estimation in the present work is based on the calculation of the *MF* in the extended alpha band (4–15 Hz), as previous EEG studies on AD recommended [29]. This is shown in the following equation:
  (3) $$\overset{IAF}{\underset{1Hz}{\sum }}PSD_{n}\left(f\right)=0.5\overset{15Hz}{\underset{4Hz}{\sum }}PSD_{n}\left(f\right).$$
- *SE* estimates the signal irregularity in terms of the flatness of the power spectrum [30]. On the one hand, a uniform power spectrum with a broad spectral content (e.g., a highly irregular signal like white noise) provides a high entropy value. On the other hand, a narrow power spectrum with only a few spectral components (e.g., a highly predictable signal like a sum of sinusoids) yields a low *SE* value. The equation for calculating *SE* would be:
  (4) $$SE=-\sum_{1Hz}^{70Hz}PSD_{n}\left(f\right)\cdot \log\left[PSD_{n}\left(f\right)\right].$$

##### Nonlinear Analysis

Alterations caused by AD and MCI also modify complexity, variability and the irregularity of the EEG activity [9,12,31,32,33,34]. Hence, to complement the spectral analysis, five global nonlinear methods were also calculated: Lempel–Ziv complexity (*LZC*), central tendency measure (*CTM*), sample entropy (*SampEn*), fuzzy entropy (*FuzzyEn*), and auto-mutual information (*AMI*).

- *LZC* estimates the complexity of a finite sequence of symbols. *LZC* analysis is based on a coarse-graining of measurements. Therefore, the EEG signal must be previously transformed into a finite symbol string. In this study, we used the simplest possible way: a binary sequence conversion (zeros and ones). By comparison with a threshold *T_d_*, the original signal samples are converted into a 0–1 sequence P=s(1), s(2), …, s(N) with s(i) defined by:
  (5) $$s\left(i\right)=\{\begin{array}{c} 0\;if\;x\left(i\right)<T_{d}\\ 1\;if\;x\left(i\right)\geq T_{d} \end{array}.$$
  The threshold *T_d_* is estimated as the median value of the signals amplitude in each channel because it is more robust to outliers. The string *P* is then scanned from left to right and a complexity counter c(N) is increased by one every time a new subsequence of consecutive characters is encountered in the scanning process. In order to obtain a complexity measure that is independent of the sequence length, c(N) should be normalized. For a binary conversion, the upper bound of c(N) is given by $b\left(N\right)=N/\log_{2}\left(N\right)$ and c(N) can be normalized via b(N):
  (6) $$LZC=\frac{c\left(N\right)}{b\left(N\right)}.$$
  *LZC* values are normalized between 0 and 1, with higher *LZC* values for more complex time series. The detailed algorithm for *LZC* measure can be found in [35].
- *CTM* quantifies the variability of a given time series on the basis of its first-order differences. For *CTM* calculation, scatter plots of first differences of the data are drawn. The value of *CTM* is computed as the proportion of points in the plot that fall within a radius *ρ*, which must be specified [36]. For a time series with *N* samples, N−2 would be the total number of points in the scatter plot that can be plotted by representing x(n+2)−x(n+1) versus x(n+1)−x(n). Subsequently, the *CTM* of the time series can be computed as:
  (7) $$CTM=\frac{\sum_{i=1}^{N-2}\delta \left(d_{i}\right)}{N-2},$$
  where
  (8) $$\delta \left(d_{i}\right)=\{\begin{array}{c} 1\;if\;{\left[{\left(x\left(i+2\right)-x\left(i+1\right)\right)}^{2}+{\left(x\left(i+1\right)-x\left(i\right)\right)}^{2}\right]}^{\frac{1}{2}}<\rho \;\\ 0\;otherwise \end{array}.$$
  Thus, *CTM* ranges between 0 and 1, with higher values corresponding to points more concentrated around the center of the plot (i.e., corresponding to less degree of variability).
- *SampEn* is an embedding entropy used to quantify the irregularity. It can be applied to short and relatively noisy time series [37]. To compute *SampEn*, two input parameters should be specified: a run length *m* and a tolerance window *r*. *SampEn* is the negative natural logarithm of the conditional probability that two sequences similar for *m* points remain similar at the next point, within a tolerance *r*, excluding self-matches [37]. Thus, *SampEn* assigns a nonnegative number to a time series, with larger values corresponding to greater signal irregularity. For a time series of *N* points, X(n)={x(1),x(2), …, x(N)}, the k=1, …, N−m+1 vectors of length *m* are formed as $X_{m}\left(k\right)=\left\{x\left(k+i\right),\;i=0,\;\ldots ,\;m-1\right\}$. The distances among vectors are calculated as the maximum absolute distance between their corresponding scalar elements. $B_{i}$ is the number of vectors that satisfy the condition that their distance is less than *r*. The counting number of different vectors is calculated and normalized as [37]:
  (9) $$B^{m}\left(r\right)=\frac{1}{N-m}\overset{N-m}{\underset{i=1}{\sum }}\frac{B_{i}}{N-m-1}.$$
  Repeating the process for vectors of length *m* + 1, $B^{m+1}\left(r\right)$ can be obtained and *SampEn* can be defined as:
  (10) $$SampEn\left(m,r\right)=-ln\left[\frac{B^{m+1}\left(r\right)}{B^{m}\left(r\right)}\right].$$
- *FuzzyEn* provides information about how a signal fluctuates with time by comparing the time series with a delayed version of itself [38]. As *SampEn*, higher *FuzzyEn* values are associated with more irregular time series. To compute *FuzzyEn*, three parameters must be fixed. The first parameter, *m*, is the length of the vectors to be compared, like in *SampEn*. The other ones, *r* and *n*, are the width and the gradient of the boundary of the exponential function, respectively [38]. Given a time series X(n)={x(1), x(2), …,x(N)}, the *FuzzyEn* algorithm reads as follows:
  1. Compose *N* − *m* + 1 vectors of length *m* such that:
    (11) $$X_{i}^{m}=\left\{x\left(i\right),x\left(i+1\right),\;\ldots ,\;x\left(i+m-1\right)\right\}-x_{0}\left(i\right),$$
    where $x_{0}\left(i\right)$ is given by:
    (12) $$x_{0}\left(i\right)=\frac{1}{m}\overset{m-1}{\underset{j=0}{\sum }}x\left(i+j\right).$$
  2. Compute the distance, $d_{ij}^{m}$, between each two vectors, $X_{i}^{m}$ and $X_{j}^{m}$, as the maximum absolute difference of their corresponding scalar components. Given *n* and *r*, calculate the similarity degree, $D_{ij}^{m}$, between $X_{i}^{m}$ and $X_{j}^{m}$ through a fuzzy function $\mu \left(d_{ij}^{m},\;n,\;r\right)$:
    (13) $$D_{ij}^{m}\left(n,r\right)=\mu \left(d_{ij}^{m},\;n,\;r\right)=\exp\left[-\frac{{\left(d_{ij}^{m}\right)}^{n}}{r}\right].$$
  3. Define the function $\phi^{m}$ as:
    (14) $$\phi^{m}\left(n,r\right)=\frac{1}{N-m}\overset{N-m}{\underset{i=1}{\sum }}\left(\frac{1}{N-m+1}\overset{N-m}{\underset{j=1,\;j\neq i}{\sum }}D_{ij}^{m}\right).$$
  4. Increase the dimension to *m* + 1, form the vector $X_{i}^{m+1}$ and the function $\phi^{m+1}$. Finally, *FuzzyEn(m*, *n*, *r*) is defined as the negative natural logarithm of the deviation of $\phi^{m}$ from $\phi^{m+1}$:
    (15) $$FuzzyEn\left(m,\;n,\;r\right)=\ln\left[\phi^{m}\left(n,r\right)\right]-\ln\left[\phi^{m+1}\left(n,r\right)\right].$$
- *AMI* is the particularization of mutual information applied to time-delayed versions of the same sequence. Mutual information is a metric derived from Shannon’s information theory to estimate the information gain from observations of one random event on another [31]. *AMI* estimates, on average, the degree to which a time-delayed version of a signal can be predicted from the original one. Thus, more predictable time series, and accordingly more regular, lead to higher *AMI* values. The *AMI* between X(n) and X(n+k) is [31]:
  (16) $$AMI=\underset{X\left(n\right),\;\;X\left(n+k\right)}{\sum }P_{XX\tau }\left[X\left(n\right),\;X\left(n+k\right)\right]\log_{2}\left\{\frac{P_{XXk}\left[X\left(n\right),X\left(n+k\right)\right]}{P_{Xk}\left[X\left(n\right)\right]\;P_{Xk}\left[X\left(n+k\right)\right]}\right\},$$
  where $P_{Xk}\left[X\left(n\right)\right]$ is the probability density for the measurement X(n), while $P_{XXk}\left[X\left(n\right),\;X\left(n+k\right)\right]$ is the joint probability density for the measurements of X(n) and X(n+k). In this study, the *AMI* was estimated over a time delay from 0 to 0.5 s and was then normalized, so that *AMI*(k=0)=1.

#### 2.3.2. Feature Selection: Fast-Correlation-Based Filter

The aforementioned characterization of the EEG may lead to the extraction of several features that provide similar information about the brain dynamics in AD, MCI, and HC. Consequently, a feature selection stage was also included. In our study, FCBF was used to discard those redundant features that share more information with the other ones than with the variable that defines the group membership. FCBF is based on symmetrical uncertainty (*SU*), which is a normalized quantification of the information gain between each feature and the group membership variables [15]. It consists of two steps: relevance and redundancy analyses of the features.

- In the first step, a relevance analysis of the features is done. Thus, *SU* between each feature *X_i_* and the group membership *Y* is computed as follows:
  (17) $$SU\left(X_{i},Y\right)=2\left[\frac{H\left(X_{i}\right)-H\left(X_{i}|Y\right)}{H\left(X_{i}\right)+H\left(Y\right)}\right],\;\;\;i=1,2,\;\ldots ,\;I,$$
  where *H*(*·*) is the well-known Shannon’s entropy, *H*(*X_i_|Y*) is the Shannon’s entropy of *X_i_* conditioned on *Y*, and *I* is the number of features extracted (in our study, *I* = 14 features). *SU* is normalized to the range [0, 1], with a value of *SU* = 1, indicating that, when knowing one feature, it is possible to completely predict the other, and a value of *SU* = 0 indicates that the two variables are independent. Then, a ranking of features is done based on their relevance since the higher the value of *SU* is, the more relevant the feature is.
- The second step is a redundancy analysis used to discard redundant features. *SU* between each pair of features *SU*(*X_i_*, *X_j_*) is sequentially estimated beginning from the first-ranked ones. If *X_i_* shares more information with *X_j_* than with the corresponding group *Y*, *SU*(*X_i_*, *X_j_*) ≥ *SU*(*X_i_*, *Y*) (with *X_i_* being more highly ranked than *X_j_*), the feature *j* is discarded due to redundancy and it is not considered in subsequent comparisons. The optimal features are those not discarded when the algorithm ends.

#### 2.3.3. Classification Approach

The described AD-MCI-HC diagnosis problem corresponds to a pattern classification task. Specifically, it can be modeled as a three-class classification problem. Bayesian decision theory establishes the rule to make such a decision to minimize the probability of misclassification [39]. We have implemented LDA, QDA, and MLP models to ensure that our conclusions take into account a variety of classification methodologies. In this study, we classify trials using each trained model, and, then, every subject is classified by means of a majority vote of all its trials [22].

##### Linear and Quadratic Discriminant Analysis (LDA and QDA)

LDA takes an input vector and assigns it to one out of the *K* classes using linear hyperplanes as decision surfaces [40]. This classifier assumes that different classes generate data based on different Gaussian distributions, whose parameters are estimated with the fitting function during the training. In order to predict the classes of new data, the trained model finds the class with the smallest misclassification cost assuming that the covariance matrices of each class are identical (homoscedasticity) [40].

QDA is a classification approach closely related to LDA. However, there is no assumption that the covariance of all classes are identical among them and it establishes a quadratic decision boundary between classes in the feature space [40].

##### Multi-Layer Perceptron Artificial Neural Network (MLP)

MLP is an artificial neural network that maps an input vector onto a set of output variables using a nonlinear function controlled by a vector of adjustable parameters. The use of neural networks for classification issues has some advantages. First, no prior assumptions about the distribution of the data are required, since neural network algorithms adjust themselves to the environment by means of the training or learning process. Thus, complex relationships can be modeled by these algorithms [41].

An MLP consists of three or more layers (an input and an output layer with one or more hidden layers) of neurons, with each layer fully connected to the next one. In our study, we have evaluated MLP networks with a single hidden layer of neurons, since networks with this architecture are capable of universal approximation [42]. MLP utilizes backpropagation in conjunction with an optimization method, such as gradient descent, with the aim of finding appropriate weights to connect neurons each other. Backpropagation is based on the definition of a suitable error function, which is minimized by updating the weights in the network [39].

In order to predict the classes for new data, the trained MLP model provides the posterior probability of belonging to each class. A three-class classification problem involves the use of three output neurons, one neuron per group. In our study, the number of neurons in the hidden layer (*n_h_*) and a regularization parameter (*u*) were optimized by cross-validation leaving all trials of a subject out in every iteration in the training set. This procedure was carried out 30 times to minimize the effect of network random initialization and then the results were averaged [43]. NETLAB toolbox was used to implement the neural network classifier [44].

### 2.4. Statistical Analysis

The three-class diagnostic ability of the models was assessed in terms of accuracy (*Acc*, overall percentage of subjects rightly classified) and Cohen’s kappa (*k*). *k* measures the agreement between predicted and observed classes, avoiding the part of agreement by chance [45]. On the other hand, the performance of the models for HC vs. all and AD vs. all comparison was described by sensitivity (*Se*, percentage of positive subjects appropriately classified), specificity (*Sp*, percentage of negative subjects correctly classified), *Acc*, positive predictive value (*PPV*, proportion of positive estimations of the models that are true positive results) and negative predictive value (*NPV*, proportion of negative estimations of the models that are true negative results).

## 3. Results

According to the proposed methods, we calculated 14 features from each EEG channel. Nine spectral features: *RP*(*d*) (where *RP*(*d*) represents de *RP* value for the *d* band), *RP*(*q*), *RP*(*a*), *RP*(*b*_1_), *RP*(*b*_2_), *RP*(*g*), *MF*, *IAF*, and *SE*, and five derived from the nonlinear methods: *LZC*, *CTM*, *SampEn*, *FuzzyEn*, and *AMI*. The results were obtained based on all the artifact-free trials within the five-minute period of recording. Results from all EEG channels were averaged in order to achieve one value per trial for each method.

### 3.1. Training Set

In order to select the optimal value of the different input parameters of each feature, only a training set was used. The optimal value for *r* (*CTM*) was obtained by evaluating the range *r* ∈ [0.01, 0.5] (step = 0.005). Values of *r* <0.01 were not considered, since they led to a *CTM* value close to 0 for every subject, whereas values of *r* >0.5 were also discarded since they led to *CTM* values equal to 1 regardless the group. For both *SampEn* and *FuzzyEn*, *m* and *r* optimal values were obtained by evaluating all the combinations for *m* = 1, 2 and *r* ∈ (0.1·SD, 0.25·SD) (step = 0.05), where SD is the standard deviation of the time series [38,46]. In the case of *FuzzyEn*, values of *n* = 1, 2, 3 were also evaluated to obtain its optimal value [38]. We chose those configurations (*r* = 0.075 for *CTM*; *m* = 1 and *r* = 0.1·SD for *SampEn*; and *m* = 1, *r* = 0.1·SD, and *n* = 3 for *FuzzyEn*) for which the corresponding *CTM*, *SampEn*, and *FuzzyEn* values showed the lowest *p*-value (Kruskal–Wallis test) among the three groups. Table 2 summarizes the averaged results for each group, taking into account only the training set. After feature extraction, FCBF was applied to the training set. The final FCBF optimal set was composed of three features: two spectral measures (*IAF* and *RP*(*d*)) and a nonlinear one (*SampEn*).

**Figure 2:**
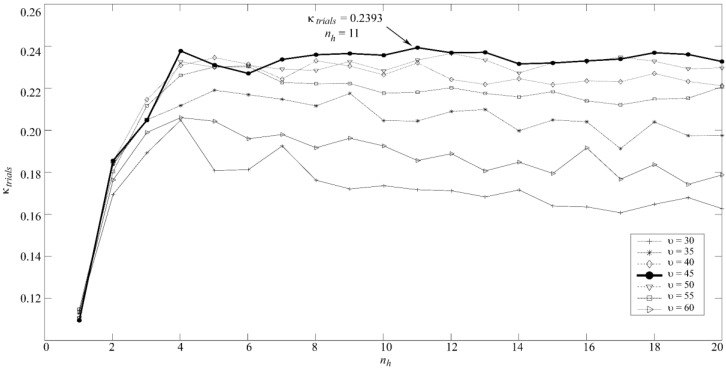
Optimal regularization parameter (*υ*) and number of neurons in the hidden layer (*n_h_*) for MLP.

The MLP model was obtained according to the optimal values for *n_h_* and *u*. Both were optimized by cross-validation, leaving all trials for each subject out in every iteration. For each value of *u* between 0 and 100 (step = 5), we varied the number of neurons in the hidden layer from 1 to 20 (step = 1) in order to compute the *k* value. This procedure was carried out 30 times to minimize the effect of network random initialization. Then, the *k* values were averaged [43]. The optimal values (highest *k* for trials) were *u =* 45 and 11 neurons in the hidden layer, as Figure 2 shows. On the other hand, since LDA and QDA models have no tuning parameters to be optimized, these were trained using all trials in the training set.

### 3.2. Test Set

Once the models were trained, their diagnostic ability was only evaluated using the test set. The overall accuracy of the models in the three-class classification task was 58.82% with LDA, 60.78% with QDA, and 62.75% with MLP. Additionally, we obtained *k* values of 0.3824 with LDA, 0.4118 with QDA and 0.4412 with MLP. These results show that MLP outperformed the discriminant analyses classifiers.

Table 3 displays the confusion matrices of each model, i.e., the model class estimation for each subject versus their actual group. As expected, the three models had higher difficulties when classifying MCI trials and subjects, as this is an intermediate state between HC and AD.

Table 4 shows *Se*, *Sp*, *Acc*, *PPV* and *NPV* for each method for HC vs. all and AD vs. all, derived from confusion matrices. MLP showed the highest diagnostic performance when determining whether a subject is not healthy (HC vs. all classification tasks: *Se* = 82.35% and *PPV* = 84.85%). Furthermore, the network showed the highest diagnostic capability when determining whether a subject does not suffer from AD (AD vs. all comparison: *Sp* = 79.41% and *NPV* = 84.38%). LDA and QDA showed similar tendencies although reaching lower diagnostic performance than MLP, as Table 4 shows.

## 4. Discussion

### 4.1. Spectral and Nonlinear Characterization of AD and MCI

Our spectral results suggested that AD and MCI elicit a slowing of spontaneous EEG activity. Further inspection of *RP* values revealed that AD patients reached higher *RP* values in low frequency bands (*q*) and lower *RP* values in high frequency bands (*b*_1_, *b*_2_ and *g*) than HC subjects. For the MCI group, a slight slowing of neural oscillations was found in comparison with HC. This increase of slow rhythms in spontaneous EEG activity was also observed by means of *MF* and *IAF*. Both spectral parameters were lower for AD patients than for MCI and HC subjects. These findings confirm the trend reported in previous studies: AD and MCI are accompanied by a progressive slow-down of EEG [24,25]. Finally, our *SE* results showed changes in the frequency distribution of the power spectrum. However, the physiological explanations for all of these alterations are not clear. The most extended hypothesis is that a significant cerebral cholinergic deficit underlies cognitive symptoms, as memory loss. A loss of cholinergic innervation of the neocortex might play a critical role in the EEG slowing associated with AD [24]. Analogously, the slowing of neural oscillations in AD could also be due to the loss of neurotransmitter acetylcholine, since the cholinergic system modulates spontaneous cortical activity at low frequencies [26].

Regarding the nonlinear parameters that quantify the complexity and irregularity of EEG recordings, our findings showed lower *LZC*, *SampEn*, *FuzzyEn* and higher *AMI* values for AD patients than for HC subjects. For these measures, MCI subjects showed intermediate values between AD and HC. Previous EEG studies also reported a loss of complexity and irregularity associated with early AD and MCI by means of nonlinear measures [9,12,31,32,33,34]. Additionally, *CTM* values were higher in AD patients and lower in HC subjects. This result suggests a decrease on variability in AD, as Abásolo et al. previously reported [12]. Taking into account the different nature of the nonlinear parameters, our results showed that the brain activity from AD patients is less complex, more regular and less variable than in MCI and HC subjects. These changes can be associated with both loss of information content and alterations in information processing at the cerebral cortex [47]. The decrease of EEG complexity can also be due to the loss of neurons or synapses, since they are associated with the complex dynamical processing within the brain neural networks [33].

### 4.2. Towards a Screening Protocol of AD

Previous studies explored several EEG features for AD and MCI discrimination from HC, focusing on binary discrimination problems (AD vs. HC, MCI vs. HC and AD vs. MCI) [16,17,18,19,20]. To the best of our knowledge, only one study performed a three-way classification, although via binary classifiers [21]. McBride et al. reached an accuracy value of 85.42% when comparing HC vs. all and 83.33% for AD vs. all (eyes closed resting condition) [21]. Although their results are slightly higher than ours (78.43% and 76.47% for both comparisons, respectively), several advantages of our methodology should be noticed. Firstly, their database was composed by only 47 subjects, in contrast to the 111 subjects recruited for our study. This data limitation also led the authors to validate its proposal through a leave-one-out cross-validation procedure instead of using a hold-out approach (training and test sets). As they obtained a different model for each iteration, the inclusion of new subjects would imply changes in every iteration of cross-validation. However, once our model is trained, the subsequent runtime to apply new data is trivial. It allows us to classify new data just feeding the trained model with the standardized version of them, simplifying the screening protocol.

In contrast to the above-mentioned studies, our MLP single model can be used not only for the three-class classification task but also in binary assessments of healthy vs. cognitively impaired subjects. As derived from Table 3 and Table 4, it has shown the ability to detect whether a subject suffers from AD or MCI in 28 out of the 34 non-healthy subjects (82.53% *Se*)—with a positive post-test probability of 84.85% (28 subjects rightly classified out of 33 subjects predicted as AD or MCI)—and only predicting two out of 17 AD patients as HC. In addition, the same model also showed the ability to discard AD in 27 out of the 34 subjects not suffering from it (79.41% *Sp*), including 15 out of the 17 HC (88.24%). These results highlight the clinical usefulness of our proposal, which might be expressed as a screening strategy similar to:

1. If the MLP model predicts AD, recommend beginning a treatment since most probably (89.47%, 17 out of 19 subjects) the patient suffers from AD or MCI.
2. If the MLP model predicts HC, do not treat the patient, since most probably (88.89%, 16 out of 18 subjects) he/she does not suffer from AD; consider a regular evaluation of the subject in the persistence of symptoms in order to minimize the number of AD and MCI missed subjects.
3. If the MLP predicts MCI, conduct a regular evaluation of the patient since doubts arise about the cognitive status of the subject.

### 4.3. Limitations and Future Research Lines

Despite the fact that we showed the usefulness of our proposal, some limitations need to be addressed. Although we used a large data sample to train and validate the models (5122 trials), they were obtained from 111 subjects. Hence, analyzing more recordings from different subjects would enhance the generalization ability of our results. Moreover, taking into account the MCI heterogeneity, it would be useful to characterize different subtypes and conduct a longitudinal analysis to characterize subjects with stable MCI and those who progress to AD. Finally, only three classification approaches (LDA, QDA, and MLP) have been used in this study. In future research works, the usefulness of other advanced classification methods, such as spiking neural networks and support vector machines, should be evaluated.

## 5. Conclusions

To sum up, our results show that both AD and MCI elicit changes in the EEG background activity: a slowing of EEG rhythms, alterations in the frequency distribution of the power spectrum, a complexity loss, a regularity increase and a variability decrease. Our proposal has shown that spectral and nonlinear features allows us to characterize the brain abnormalities associated with AD and MCI. In addition, we have shown the high diagnostic ability of different three-class models trained with this EEG information, particularly when predicting AD and HC status. These results highlight the usefulness of our proposal in order to help physicians classify AD, MCI and HC from EEG data.

## Figures and Tables

**Table 1:** Social-demographic and clinical data for each group.

	Training Set			Test Set		
	HC	MCI	AD	HC	MCI	AD
Number of subjects	20	20	20	17	17	17
Number of trials	912	937	917	752	847	757
Age (years)	75.6	77.9	80.7	76.4	75.3	82.4
(median [IQR])	[74.1, 77.6]	[67.9, 79.8]	[74.7, 83.3]	[73.6, 78.9]	[69.8, 82.0]	[77.7, 83.9]
Gender (Male:Female)	8:12	8:12	5:15	4:13	8:9	7:10
MMSE ^1^	29	27.5	21	29	27	22
(median [IQR])	[28, 30]	[26.5, 29]	[18.5, 22.5]	[28, 30]	[27, 28]	[20, 24]
B-ADL ^2^	1.1	2.9	5.8	1.2	2.8	6.4
(median [IQR])	[1.0, 1.2]	[2.4, 3.3]	[5.1, 7.2]	[1.0, 1.3]	[2.3, 2.5]	[5.0, 4.3]
Education level (A:B) ^3^	5:15	11:9	8:12	5:12	12:5	10:7

^1^ MMSE: Mini Mental State Examination; ^2^ B-ADL: Bayer-Activities of Daily Living; ^3^ A: primary education or below, B: secondary education or above.

**Table 2:** Averaged results (median (interquartile range)) for each group and for each feature taking into account only the training set.

Features	HC	MCI	AD
*RP(δ)*	0.227 [0.179, 0.277]	0.164 [0.102, 0.221]	0.158 [0.103, 0.229]
*RP(θ)*	0.111 [0.083, 0.131]	0.122 [0.087, 0.155]	0.143 [0.103, 0.188]
*RP(α)*	0.243 [0.174, 0.291]	0.317 [0.224, 0.544]	0.279 [0.192, 0.447]
*RP*(*β*_1_)	0.128 [0.101, 0.155]	0.101 [0.081, 0.160]	0.101 [0.073, 0.141]
*RP*(*β*_2_)	0.111 [0.084, 0.138]	0.105 [0.048, 0.135]	0.091 [0.060, 0.119]
*RP(γ)*	0.097 [0.074, 0.168]	0.087 [0.037, 0.145]	0.089 [0.047, 0.141]
*MF*	10.584 [9.690, 11.900]	10.467 [8.639, 12.285]	9.971 [9.030, 10.997]
*IAF*	9.502 [8.751, 9.996]	9.404 [8.519, 9.972]	8.811 [8.510, 9.474]
*SE*	0.813 [0.760, 0.822]	0.796 [0.695, 0.816]	0.782 [0.733, 0.809]
*LZC*	0.684 [0.6331, 0.7360]	0.667 [0.551, 0.731]	0.663 [0.589, 0.713]
*CTM*	0.101 [0.076, 0.129]	0.111 [0.086, 0.165]	0.116 [0.077, 0.183]
*SampEn*	1.366 [1.288, 1.540]	1.312 [1.103, 1.489]	1.274 [1.034, 1.489]
*FuzzyEn*	0.532 [0.466, 0.624]	0.514 [0.395, 0.618]	0.508 [0.427, 0.584]
*AMI*	−0.149 [−0.184, −0.130]	−0.149 [−0.175, −0.124]	−0.145 [−0.164, −0.128]

**Table 3:** Confusion matrices of each model: trials and subjects’ classification in the test set.

	LDA			QDA			MLP		
Actual ↓\Estimated →	HC	MCI	AD	HC	MCI	AD	HC	MCI	AD
HC	11	4	2	13	3	1	12	3	2
MCI	4	7	6	4	7	6	4	8	5
AD	2	3	12	3	3	11	2	3	12

**Table 4:** Diagnostic performance for HC vs. all and AD vs. all, derived from confusion matrices.

	HC vs. All			AD vs. All		
	LDA	QDA	MLP	LDA	QDA	MLP
*Se* (%)	82.35	79.41	82.35	70.59	64.71	70.59
*Sp* (%)	64.71	76.47	70.59	76.47	79.41	79.41
*Acc* (%)	76.47	78.43	78.43	74.51	74.51	76.47
*PPV* (%)	82.35	87.10	84.85	60.00	61.11	63.16
*NPV* (%)	64.71	65.00	66.67	83.87	81.82	84.38
